# Effects of habitat and time of day on flock size of Turkey Vultures in Cuba (*Cathartes aura*)

**DOI:** 10.3897/zookeys.726.14581

**Published:** 2018-01-08

**Authors:** Piotr Tryjanowski, Federico Morelli

**Affiliations:** 1 Institute of Zoology, Poznań University of Life Sciences, Wojska Polskiego 71 C, 60-625 Poznań, Poland; 2 Czech University of Life Sciences Prague, Faculty of Environmental Sciences, Department of Applied Geoinformatics and Spatial Planning, Kamýcká 129, CZ-165 00 Prague 6, Czech Republic

**Keywords:** Cuba, large-scale survey, roadside, scavenger, vulture

## Abstract

In agricultural landscapes, the Turkey Vulture *Cathartes aura* feeds mainly on carcases of domestic animals. In spring 2017, data on 214 flocks of Turkey vultures were collected in a road survey in Cuba (in total 2384 km). Turkey Vultures were found to be common accross Cuba, but flock size varied between habitats, reaching a maximum of 43 in valleys and 31 in agricultural landscapes with domestic animal farms. Vultures were active throughout the day, but the time of day did not significantly affect flock size. This study corroborates previous studies which suggested that carrion resources located in agricultural habitats and river valleys is crucial for the continued survival of this still abundant species. Changes in Cuba’s socio-political system in the near future will likely impact agricultural practices, and this in turn will likely affect Turkey Vultures. Our study may serve as a baseline against which future population changes and flocking behaviour of Turkey Vultures can be compared.

## Introduction

Vultures are globally endangered birds (Ogada et al. 2012). Vultures play significant roles in the environment and have interacted with humans over long periods of time (Morelli et al. 2015). Recently, several species of vultures have shown steep declines in population size. However, some species of vultures still have seemingly healthy populations. One such species is the Turkey vulture *Cathartes aura* (Linnaeus, 1758), which is found in North, Central and South America, including the Caribbean islands (Kirk and Mossman 1998). The species is common in Cuba, where it is the most abundant raptor species recorded (Wotzkow and Wiley 1988, Ferrer-Sánchez and Rodriguez-Estrella 2014). This presents an opportunity to investigate the reasons for the success of this species in comparison to other vultures which are in danger of extinction. One strategy to understand vulture declines is to study access to healthy food and behaviour in relation to foraging capability, including the formation of flocks, habitat use, and time-activity pattern (Ogada et al. 2012). However, all these parameters are susceptible to rapid environmental changes caused by changing agricultural and urban policies, including use of medical products, such as the anti-inflammatory drug diclofenac (Camiña and Yosef 2012, Balmford 2013). Cuba is just beginning to transform its urban and rural landscapes, which is associated with the socio-political changes of recent years (Graddy‐Lovelace 2017).

The aims of this study were to (1) assess the influence of habitat variation on flock size, (2) describe perches preferred by Turkey Vultures, and (3) assess how the time of the day affects flocking behaviour of the vultures. Results are discussed in the light of the changes expected to occur in the agricultural systems of Cuba due to recent socio-political changes, and which may affect bird populations in a way similar to what occurred in post-communist Europe (Tryjanowski et al. 2011, Batáry et al. 2017).

## Methods

**Figure 1.**
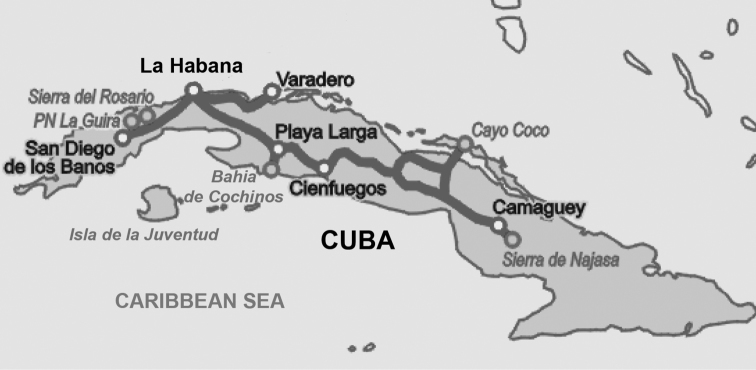
The map of Cuba with indicated (grey line) map of the transect.

Surveys were conducted from March to April 2017 along roads throughout Cuba (in total 2384 km; Figure 1) using methods described by Wuczyński (2001) and Watson and Simpson (2014). One or two observers, passenger(s), scanned power lines, utility poles, trees, fences, buildings, open fields, and the skyline for vultures, and identified the birds using binoculars. Species, the number of individuals, location, time, and habitat for all vultures were recorded. Groups of vultures flying or sitting together were defined as a flock (cf. Kirk and Mossman 1998, Jackson et al. 2008).

Search effort was determined by habitat availability from the roads. Habitats were classified according to Ferrer-Sánchez and Rodríguez-Estrella (2015): (1) open fields and meadows; (2) valleys in mountains connected mainly with rivers; (3) waters, including also coastlines, lagoons, and swamp marsh; (4) villages, and (5) urban habitats including hotels and their infrastructure.

The associations among flock size, habitat type and time of day were assessed by means of a linear model procedure. Flock size was entered as response variable while time and type of habitat were used as predictors. The model was fitted assuming a log-normal distribution of response variables after having explored the distribution of these variable as suggested by Box and Cox (1964) using the packages ‘MASS’ (Venables and Ripley 2002), and ‘glmmADMB’ in R (Fournier et al. 2012, Skaug et al. 2013). The full model was based on 214 observations. Confidence intervals for the significant variables were calculated by the Wald method from the package ‘MASS’ (Venables and Ripley 2002). All statistical tests were performed in R (R Development Core Team 2017).

## Results

### Flock sizes

**Figure 2.**
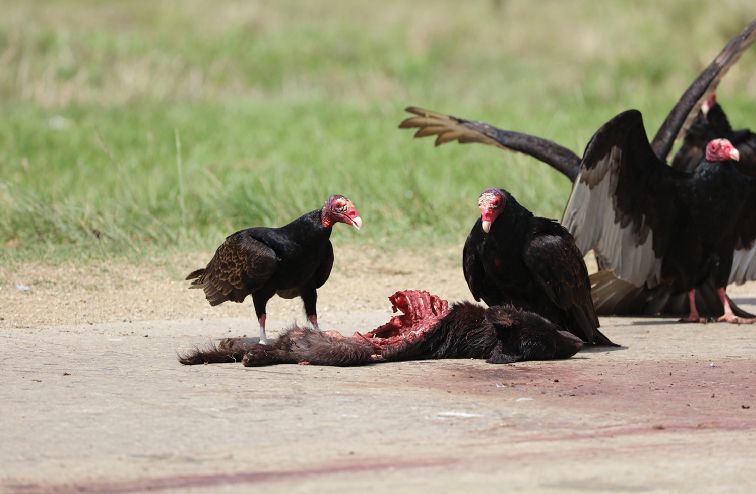
Turkey vulture foraging on a road-kill dog, in a village on the north from Moron, province Ciego de Ávila (photograph P. Tryjanowski).

**Figure 3.**
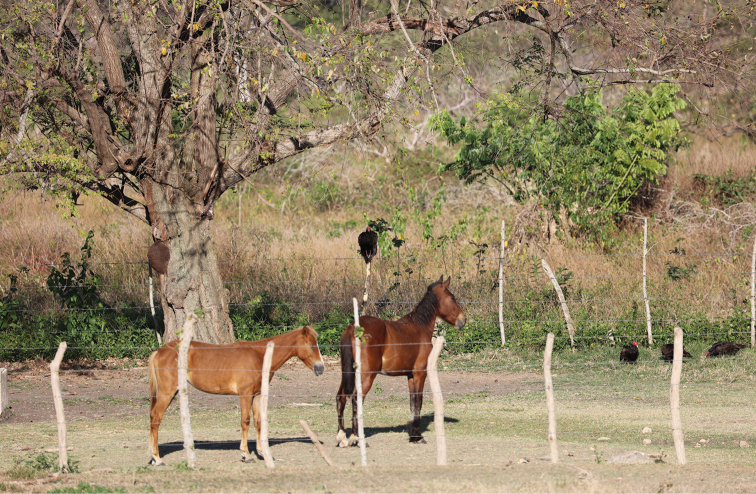
Perched Turkey vultures on a horse farm, Sierra del Chorrillo, province Camagüey (photograph P. Tryjanowski).

**Figure 4.**
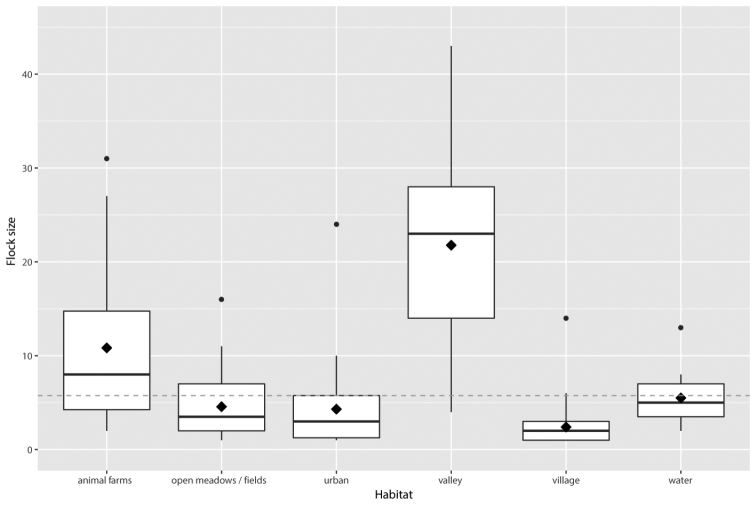
Flock size of Turkey Vultures in relation to habitat type. The y-axis represents the estimated variable. The boxplots show the median (bar in middle of rectangles), mean (black rhombus), upper and lower quartiles, and extreme values. The horizontal dashed line is the average values of flock sizes considering all cases.

**Figure 5.**
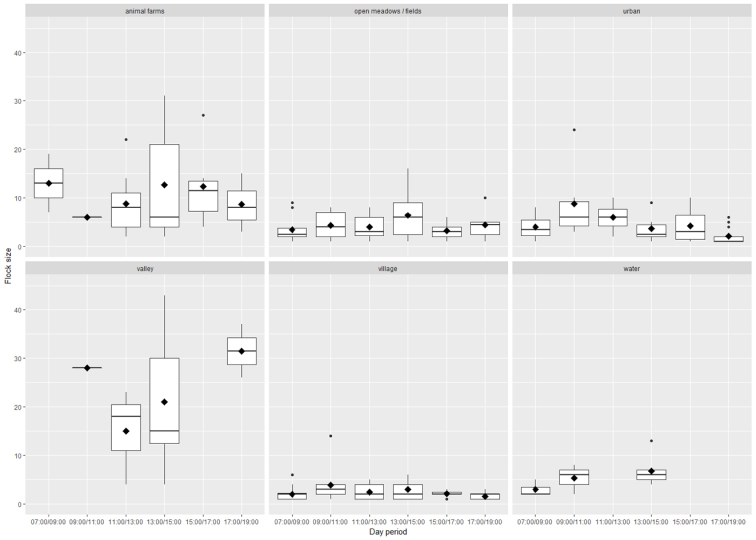
Flock size of Turkey Vultures in relation to time of day. The y-axis represents the estimated variable. The boxplots show the median (bar in middle of rectangles), mean (black rhombus), upper and lower quartiles, and extreme values.

In total, 1231 individuals in 214 flocks were observed during the study period. Habitat influenced average flock size, with the largest flocks recorded in valleys (43 individuals) and in the vicinity of domestic animal farms (31 individuals) in an agricultural landscape (Figures 2 and 3). Differences in flock size between habitats were statistically significant (Table 1). The flock size of vultures was significantly smaller in urbanised areas (urban areas and villages) than in agricultural landscapes (Figure 4, Table 1). Although flock size strongly varied with time of day, there was no significant relationship between flock size and time of day, nor between flock size and the interaction of time with habitat (Table 1, Figures 4 and 5).

**Table 1.** Relationship between flock size of Turkey Vultures to habitat type and time of day (N = 214). Abbreviations: 2.5%, lower level of confidence interval; 97.5%, upper level of confidence interval; SE, standard error. The R^2^ for the model multiple was 0.419.

Variable	Estimate	2.5%	97.5%	SE	t	p
(Intercept)	2.304	1.780	2.828	0.266	8.669	< 0.05
Habitat (open field)	-0.900	-1.250	-0.549	0.177	-5.064	< 0.05
Habitat (urban)	-1.002	-1.354	-0.649	0.178	-5.609	< 0.05
Habitat (valley)	0.774	0.284	1.263	0.248	3.116	< 0.05
Habitat (village)	-1.457	-1.779	-1.136	0.163	-8.924	< 0.05
Habitat (water)	-0.582	-1.091	-0.074	0.258	-2.260	< 0.05
Time	-0.014	-0.049	0.017	0.016	-0.886	n.s.

### Perch sites

A total of 57 perched birds was observed: 32 (56.1%) on trees, eleven (19.3%) sitting on the ground, eight (14.0%) on electric pylons, and six (10.5%) on fences (Figure 3).

## Discussion

Turkey vultures feed heavily on carrion of domestic animals in landscapes dominated by agriculture (Kirk and Mossman 1998, Ogada et al. 2012). On the other hand, Turkey vultures are also recognised as foraging opportunists, making use of various food sources, including vegetables (Kelly et al. 2007, Platt and Rainwater 2009).

In our study, we found the largest flocks near domestic animal farms and in river valleys, where probably carrion of large animals, both wild and domestic, is frequently available. In agreement with previous studies, relatively small numbers of vultures were observed in non-agricultural landscapes. This pattern of flock size related to habitat suggests that the availability carrion of domestic animals is crucial for the vultures’ presence and abundance (Camiña and Yosef 2012, Ogada et al. 2012, Balmford 2013). Perched vultures were noted less often than flying birds (only 4.6% of all recorded individuals). This can be partially attributed to survey methods and differences in detectability of perched and flying vultures (Watson and Simpson 2014).

Flying vultures are often noted throughout the day (Mandel and Bildstein 2007); however, no strong pattern emerged from the data. There was a tendency towards larger flock in the afternoon when thermals were stronger and vultures could extend their daily activity period, especially in urban habitats, as was suggested also by Mandel and Bildstein (2007).

During this study, we confirmed previous findings that the Turkey Vulture is still very common in Cuba (Wotzkow and Wiley 1988, Ferrer-Sánchez and Rodríguez-Estrella 2015). However, due to expected socio-economical changes (Ferrer-Sánchez and Rodríguez-Estrella 2015, Graddy‐Lovelace 2017) changes in bird populations can be very rapid (Balmford 2013), as were shown, for example, in farmland birds in eastern Europe since the early 1990s (Tryjanowski et al. 2011). Therefore, to conserve the best patches of habitat for vultures in Cuba, it is crucial to identify the appropriate habitats today and implement protection measures in order to ensure healthy populations in the future.
